# Effects of bathing-induced changes in body temperature on sleep

**DOI:** 10.1186/s40101-023-00337-0

**Published:** 2023-09-08

**Authors:** Takafumi Maeda, Hiroko Koga, Takashi Nonaka, Shigekazu Higuchi

**Affiliations:** 1 Department of Human Life Design and Science, Faculty of Design, Kyushu University, 4-9-1, Shiobaru, Minami-Ku, Fukuoka, 815-8540 Japan; 2 Physiological Anthropology Research Center, Faculty of Design, Kyushu University, 4-9-1, Shiobaru, Minami-Ku, Fukuoka, 815-8540 Japan; 3 Research & Development Division, Noritz Corporation, 5, Minami-Futami, Futamicho, Akashi, Hyogo 674-0093 Japan

**Keywords:** Bathing, Body temperature, Sleep onset latency, Sleep quality, Distal-to-proximal skin temperature gradient

## Abstract

**Background:**

Passive body heating before sleep is well known to lead to improved sleep. However, the effects of the degree of change in body temperature by bathing on sleep quality are unclear. The present study aimed to clarify the effects on sleep of bathing-induced changes in body temperature.

**Methods:**

Twenty-three healthy males and females in their 20 s to 50 s bathed in their homes 1.5–2 h before bedtime under three bathing conditions: showering only; short bathing in a bathtub; and long bathing in a bathtub. Sublingual and skin temperatures and thermal sensation before and after bathing, sleep indices such as sleep onset latency, time in bed, sleep efficiency, and wake after sleep onset, all of which were evaluated using an actimeter, and subjective evaluations of sleep were compared among conditions.

**Results:**

Sublingual temperature just after bathing was significantly higher with long bathing than with other conditions, and the fall in sublingual temperature from after bathing to before sleep was significantly larger with long bathing than with short bathing. Sleep onset latency by actimeter was significantly reduced with long bathing compared to showering. In addition, subjective evaluations of falling asleep and sleep quality were better with long bathing than with showering or short bathing.

**Conclusions:**

In conclusion, bathing conditions that produce a 0.9 °C increase in sublingual temperature appear effective for falling asleep and sleep quality, because core temperature shows a greater drop to before sleep than those producing an increase of about 0.3 °C increase in sublingual temperature.

## Background

Many people, especially working age individuals in their 20 s to 50 s, are dissatisfied with sleep [1]. Sleep is disturbed by various factors, one of which sleep onset latency (SL) is reportedly prolonged in persons with low skin temperature in the peripheral extremities [2]. Bathing is known to have positive effects on sleep, and various studies have attempted to clarify the relationship between bathing and sleep-related variables [3–12].

The effects of bathing timing on sleep have been examined in a number of investigations. Bunnell et al. [4] investigated the effects on sleep of the timing of bathing, comparing bathing in the morning (within 1 h of waking), afternoon (10 h before bedtime), evening (6 h before bedtime), and late night (immediately before bedtime). SL was shown to be shortened by bathing in the evening [4]. Inagaki et al. [8] reported that bathing 2 h before bedtime was more effective in promoting sleep quality than bathing 0.5 or 1 h before bedtime. Some studies have also reported that bathing less than 2 h before bedtime, such as 1.75 to 2 h [6] or 1.5 h [5] before bedtime, has positive effects on sleep.

A systematic review by Haghayegh et al. [13] found that passive body warming by a hot shower, foot bath for 10 min or more, or whole-body bathing within about 1–2 h before bedtime resulted in shorter SL, increased sleep efficiency, and improved subjective sleep quality. However, that review included 17 studies on footbath and sleep, but only 6 studies on whole-body bathing and sleep. Four of those 6 studies with whole-body bathing targeted the elderly, and the remaining 2 studies targeted only young people in their 20 s [3, 9]. Expanding the target group from young people to middle-aged people is therefore necessary, because improving sleep quality in working age individuals in their 20 s to 50 s is inducing the increased productivity [14–16]. In addition, although many studies have compared bathing and non-bathing conditions [6, 10], comparisons between different bathing (e.g., showering) conditions appear warranted, given the bathing habits of the young to middle-aged Japanese population, in which the ratio of showering is high [17].

Many of the studies on bathing and sleep that have reported positive effects on sleep from bathing before going to bed have been laboratory investigations [3, 6, 9, 10]. In laboratory experiments, overnight polysomnography can be used to evaluate sleep and thus can be used as an index, but the novel sleep environment itself may affect sleep. On the other hand, studies conducted in living spaces of participants have often evaluated sleep subjectively [11, 18, 19], and objective evaluations of sleep in actual living spaces are therefore needed. Kanda et al. [18] evaluated sleep objectively using a body motion sensor placed under the bed for young and elderly people in actual living spaces, revealing that bathing improved sleep quality compared with non-bathing. Tai et al. [11] conducted a cross-sectional evaluation of the effects of bathing on sleep using an actimeter in an actual living space, and clarified that bathing 60–180 min before bedtime shortens sleep onset latency. However, those previous studies did not control for bathing conditions in actual life and were unable to clarify the effects on sleep of bathing-induced changes in body temperature.

According to Haghayegh et al. [13], SL is related to the magnitude of the decrease in core temperature after body heating by bathing etc., rather than any specific value of core temperature itself due to body heating before going to bed. In other words, a greater decrease in core temperature after bathing appears important for improving SL. However, a systematic review by Haghayegh et al. [13] included no descriptions of the effects on sleep of differences in bathing time or degree of increase in core temperature due to bathing.

Regarding rises in body temperature due to bathing, a study by Dorsey et al. [6] on elderly women with insomnia found that bathing to the mid-thorax level in 40–40.5 °C water for 30 min between 1.75 and 2 h before bedtime increased rectal temperature by 0.71 ± 0.31 °C. They reported that under those bathing conditions, half of the participants showed an increase in sleep maintenance (SM), a reduction in wake after sleep onset (WASO), and an increase in slow-wave sleep compared to a night without bathing. In a study by Mishima et al. [10] on elderly people with vascular dementia, core temperature decreased by an average of about 0.8 °C from immediately after bathing to the mid-thorax level in 40 °C water for 30 min at 2 h before bedtime to bedtime and SL was significantly reduced under bathing conditions compared to the baseline night without bathing. These studies indicate a relationship between the degree of decrease in core temperature after bathing and sleep. However, the effects on sleep of the difference in core temperature rise associated with bathing have not been clarified.

The purpose of the present study was to clarify the effects of the degree of increase in core temperature with bathing on SL (falling asleep) and sleep quality using field experiments in actual life spaces, targeting a wide range of age groups from young to middle-aged.

## Methods

### Participants

Twenty-three healthy adults with regular sleeping habits participated in this study (12 males, 11 females; mean [± standard deviation] age, 39.3 ± 10.4 years; height, 165.6 ± 12.2 cm; mass, 59.0 ± 11.7 kg; body mass index, 21.4 ± 2.3 kg/m^2^). Participants ranged in age from 23 to 54 years, with 6 participants in their 20 s, 5 in their 30 s, 7 in their 40 s, and 5 in their 50 s. Shift or night workers, and individuals taking medications for sleep disturbance were excluded from the present study. Written informed consent was obtained from all participants prior to enrolment in the study. All research protocols were approved by the Ethics Committee of Noritz Corp (approval no. 0014). Participants abstained from heavy work, strenuous exercise, and alcohol consumption from the day before the experiment, abstained from smoking and napping on the day of the experiment, and abstained from caffeine intake from 18:00 prior to the scheduled tests.

According to a preliminary questionnaire, 18 participants reported trouble or dissatisfaction with sleep. Mean score for the Japanese version of the Pittsburgh Sleep Quality Index [20] was 6.3 ± 2.0, and 18 participants showed scores ≥ 5. In addition, 14 participants reported frequently feeling that their hands and/or feet were cold.

For female participants, the stage of the menstrual cycle (follicular or luteal phase) was not controlled for, but was gauged based on a questionnaire. We confirmed that the number of participants in each stage of the menstrual cycle did not differ significantly between conditions.

### Experimental environment and condition

The experiment was conducted in the home of each participant, to eliminate the effects of unfamiliar environments in the artificial climatic chamber on sleep. The study was performed from December 2020 to March 2021.

A systematic review [13] confirmed that the increase in peripheral skin temperature of the hands and feet due to bathing and the decrease in core temperature after bathing were associated with shorter SL. This study therefore set the following three conditions: a showering condition (showering) in which neither skin temperature nor core temperature rose easily; a short bathing condition in a bathtub (short bathing) in which skin temperature rose and core temperature rose slightly; and a long bathing condition (long bathing) in which both skin temperature and core temperature rose.

Under the showering condition, only the face, body, and hair were washed in the shower. Under the bathing conditions, the face, body, and hair were washed by showering, followed by full-body bathing with water level up to the mid-thorax level in a long sitting position. Water temperature in the shower and bathtub was set at about 40 °C under all conditions [5, 6, 8, 10].

The rise in core temperature due to bathing differs depending on the bathing environment and individual characteristics [5, 21, 22], even when bathing time is the same. In the present study, bathing time was determined in advance for each participant with a target rise in rectal temperature of about 0.5 °C in the long bathing condition and about 0.1 °C in the short bathing condition, which based on a predicted rectal temperature change using the thermal model for bathing [23]. The target rise of about 0.5 °C was set, in consideration of safety, referring to a previous study in which core temperature rose by about 0.7 °C [6, 10, 23]. Bathing duration was 5.2 ± 1.4 min for short bathing and 16.1 ± 3.0 min for long bathing.

Bathing was started 1.5–2 h before going to bed, based on previous studies [5, 6, 8]. Air temperature in the bathroom was not controlled, but did not differ significantly among conditions, at 17.3 ± 4.8 °C for showering, 18.2 ± 4.2 °C for short bathing, and 17.5 ± 4.7 °C for long bathing.

Each participant wore their own same sleep clothes and socks from after bathing until going to bed. Participants were instructed that should be no difference in the use of heating appliances such as hot carpets and floor heating after bathing under the three conditions, and that they should go to bed and wake up at their usual times. Participants were also restricted from using electric blankets while sleeping. Air temperature in the bedroom was not controlled, but did not differ significantly between conditions, at 17.4 ± 3.2 °C for showering, 17.8 ± 3.0 °C for short bathing, and 17.8 ± 3.5 °C for long bathing.

### Experimental procedure

The experiments were conducted in the home of the participant on the night of a working day, excluding holiday or days before holidays. The three bathing conditions were applied in random order. The experiment for the first bathing condition was conducted for 4 days within 1 week, with the first day set as the acclimation day and the next 3 days as observation days. The experiment for the second and third conditions was conducted for 3 days within 1 week for each condition as observation days. Among the observation days, 1 day that satisfied the conditions for bathing timing and temperature was used for analysis.

### Measurements

#### Sleep parameters

Activity during sleep was recorded using an actimeter (FS-770; Acos, Japan) attached to the waist to objectively evaluate sleep states from posture changes during sleep. Sleep indices such as SL, time in bed (TIB), total sleep time (TST), sleep efficiency (SE), and WASO were estimated from activity data using a sleep–wake rhythm app (SleepSign Act version 2.0; Kissei Comtec, Japan) [24]. SE was calculated by formula SE = TST/TIB. The concordance rate between this system and polysomnography for sleep/wake judgment has been reported as 88.4% [24].

#### Subjective feelings of falling asleep and quality of sleep

In terms of subjective feelings regarding sleep, falling asleep and quality of sleep were measured by the semantic differential method using a 9-point scale (from 9, “very good” to 1, “very bad”) after waking.

#### Oguri-Shirakawa-Azumi sleep inventory

Subjective sleep quality was also assessed using the Oguri-Shirakawa-Azumi Sleep Inventory Middle aged and Aged version (OSA-MA) [25] the morning after each experimental night. The OSA-MA is a standardized sleep inventory comprising 16 items, answered using a 4-point scale. The 16 items can be categorized into the following five factors: Factor I, “sleepiness on rising"; Factor II, “initiation and maintenance of sleep"; Factor III, “frequent dreaming"; Factor IV, “refreshing"; and Factor V, “sleep length”. For all factors, a higher score indicates better sleep. The OSA-MA is commonly used in sleep studies of Japanese participants [26–31].

#### Sublingual and skin temperatures

Sublingual temperature was measured before and after bathing, and before sleep using a basal body thermometer (MC-652LC; Omron, Japan) to confirm changes in core temperature.

Skin temperatures at the trunk (forehead, chest) and peripheral parts (back of the hand and instep) of the body were measured before and after bathing and before sleep using an infrared thermometer (S-708; Seastar, Japan) to check changes in skin temperature. Distal-to-proximal skin temperature gradient (DPG) is an indirect index of vasodilation in the distal skin [4] that is reportedly associated with SL [32], and was calculated by subtracting chest skin temperature from the instep skin temperature.

#### Thermal sensations before sleep

Thermal sensations considering overall body and local sensations in the hands and feet were measured using a 7-point scale (7, very hot; 6, hot; 5, warm; 4, neutral; 3, cool; 2, cold; and 1, very cold) before going to sleep.

### Data screening and analysis

The following days were excluded from analysis: the first day of the experiment (the acclimation day); any day in which bathing time started within 1.5 h or 2 h or more before bedtime; and days on which the participant experienced extreme stress or poor physical condition (headache, etc.). In addition, the following days were prioritized, and one day was analyzed for each condition: for the same participant (between conditions), days after the second day of each condition; days with almost the same times of bedtime and wake-up time, and days with the same workplace (office/home).

Statistical analyses were performed using SPSS version 22.0 (IBM SPSS Statistics, USA). The Shapiro–Wilk test was performed to confirm the normality of data. In terms of physiological measurements with confirmed normality such as body temperature, blood pressure, and heart rate, two-way analysis of variance (ANOVA) with repeated measures was used to examine the main effects of condition and time, as well as interactions between both, followed by post-hoc testing using Bonferroni correction.

In terms of each sleep index obtained from the actimeter, total score and each factor score on the OSA-MA and sleep-related subjective measures for which normality was not confirmed, the Friedman test was used to examine the main effect of conditions, and the Wilcoxon test with Bonferroni correction was used to detect differences between each condition as post-hoc testing.

Value of *p* < 0.05 were considered statistically significant. All data are expressed as mean ± standard deviation.

## Results

### Sleep parameters

Bedtimes did not differ significantly among conditions, at 0:27 ± 1 h 10 min for showering, 0:26 ± 1 h 6 min for short bathing, and 0:29 ± 1 h 6 min for long bathing. Times out of bed likewise did not differ among conditions, at 6:57 ± 33 min for showering, 6:58 ± 42 min for short bathing, and 6:50 ± 34 min for long bathing.

**Fig. 1:**
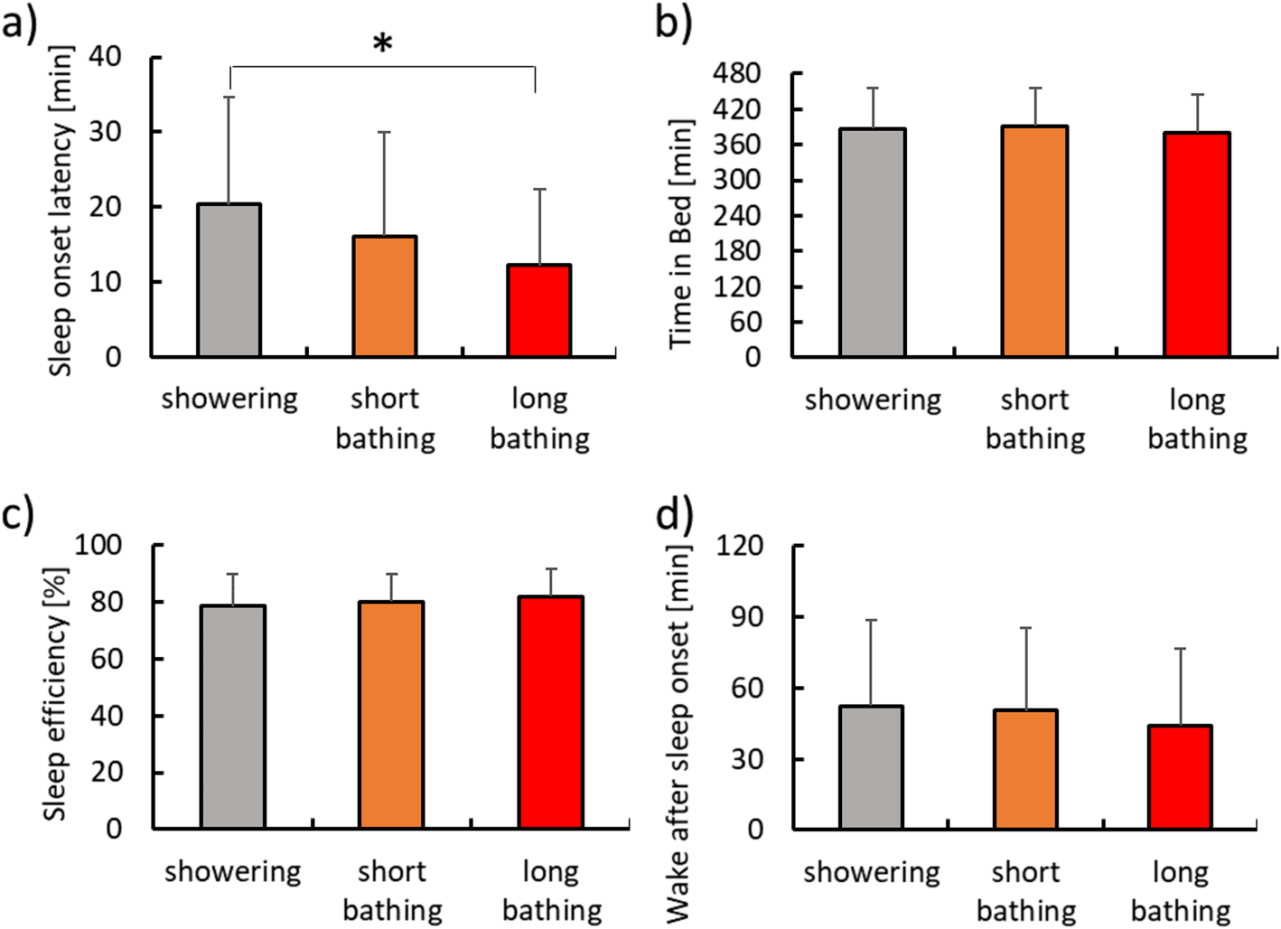
Sleep parameters assessed using the actimeter. **a** Sleep onset latency; **b** time in bed; **c** sleep efficiency; and **d** wake after sleep onset. *Significant difference, *p* < 0.05

Figure 1 indicates the results of sleep parameters assessed using the actimeter. SL was 20.3 ± 14.3 min with showering, 16.1 ± 13.9 min with short bathing, and 12.3 ± 10.1 min with long bathing (Fig. 1a). SL was significantly shorter with long bathing than with showering (*p* < 0.05). TIB was 389.5 ± 67.6 min with showering, 392.5 ± 61.9 min with short bathing, and 381.6 ± 62.4 min with long bathing (Fig. 1b). No significant differences in TIB were seen among conditions. SE was 78.69 ± 11.06% with showering, 80.30 ± 9.75% with short bathing, and 82.05 ± 9.60% with long bathing (Fig. 1c). No significant differences in SE were seen among conditions. WASO was 52.3 ± 36.4 min with showering, 50.5 ± 34.8 min with short bathing, and 44.1 ± 32.4 min with long bathing (Fig. 1d). No significant differences in WASO were seen among conditions.

### Subjective feeling of falling asleep and quality of sleep

**Fig. 2:**
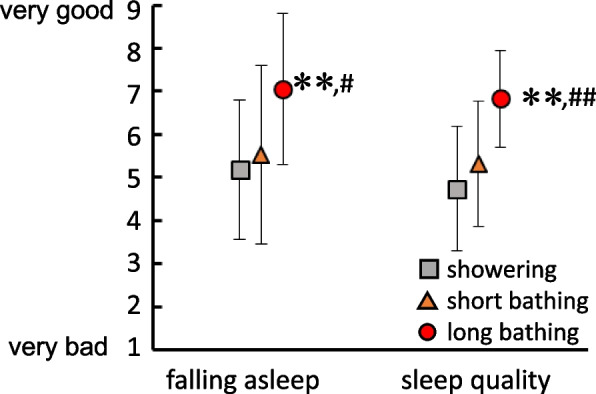
Subjective feeling of falling asleep and quality of sleep. Squares, triangles, and circles indicate showering and short and long bathing conditions, respectively. ** Significant difference from showering condition, *p* < 0.01. # Significant difference from short bathing condition, *p* < 0.05. ## Significant difference from short bathing condition, *p* < 0.01

Figure 2 indicates the results from subjective evaluations of falling asleep and quality of sleep. Falling asleep and quality of sleep were significantly better with long bathing than with other conditions (falling asleep: vs showering, *p* = 0.003; vs short bathing, *p* = 0.024; sleep quality: vs showering, *p* = 0.001; vs short bathing, *p* = 0.002).

### OSA-MA sleep score

**Fig. 3:**
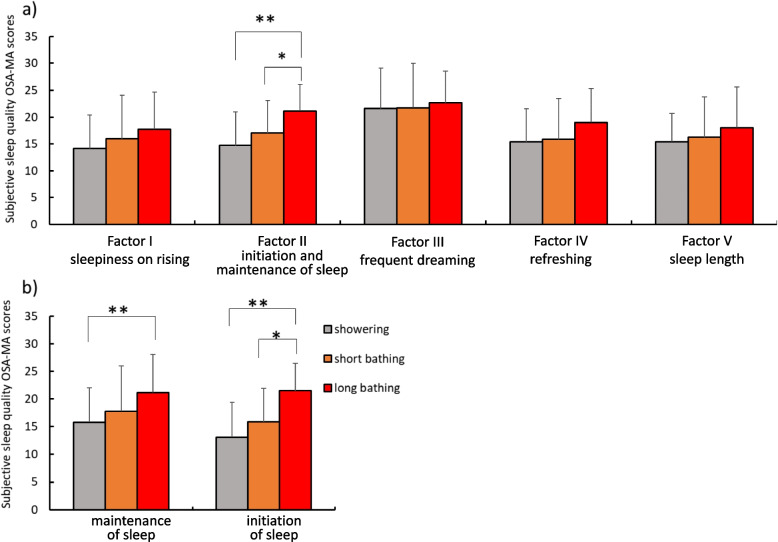
OSA-MA score. **a** Five factors of OSA-MA. **b** Contents in Factor II (initiation and maintenance of sleep). OSA-MA, Oguri–Shirakawa–Azumi Sleep Inventory, Middle-aged and Aged version. Grey, orange, and red marks indicate showering and short and long bathing conditions, respectively. *Significant difference, *p* < 0.05. **Significant difference, *p* < 0.01

Figure 3 indicates each factor for OSA-MA (Fig. 3a) and each item in Factor II, which is related to the initiation and maintenance of sleep (Fig. 3b). In terms of each factor for OSA-MA, no differences were seen in Factors I, III, IV, or V, but significant differences in scores were seen for Factor II (*p* < 0.001), with significantly higher scores for long bathing than for other conditions (vs showering, *p* < 0.001; vs short bathing, *p* = 0.045) (Fig. 3a). Concerning the contents of Factor II, SM score differed significantly between conditions (*p* = 0.001) by ANOVA, and post-hoc testing showed higher scores with long bathing than with showering (*p* = 0.002) (Fig. 3b). Sleep onset score for Factor II differed significantly between conditions (*p* < 0.001) and post-hoc testing showed significantly higher scores with long bathing than with other conditions (vs showering, *p* = 0.001; vs short bathing, *p* = 0.024) (Fig. 3b).

### Sublingual and skin temperatures and DPG

Table 1 shows sublingual temperature, skin temperatures (forehead, chest, back of the hand, and instep) and DPG before and after bathing and before sleep, changes from before to after bathing, and changes from after bathing to before sleep.

**Table 1:** Changes in sublingual and skin temperatures and DPG(°C)

Part	Conditions	Before bathing (A)	After bathing (B)	Before sleep (C)	B-A	C-B
Sublingual	Showering Short bathing Long bathing	36.48 ± 0.36 36.55 ± 0.28 36.34 ± 0.38^#^	36.83 ± 0.34 36.82 ± 0.30 37.25 ± 0.45**^,##^	36.17 ± 0.34 36.28 ± 0.35 36.31 ± 0.22	0.35 ± 0.32 0.27 ± 0.23 0.91 ± 0.40**^,##^	− 0.66 ± 0.23 − 0.55 ± 0.26 − 0.94 ± 0.49^##^
Forehead	Showering Short bathing Long bathing	32.83 ± 0.91 32.94 ± 0.97 32.49 ± 1.09	33.03 ± 1.12 33.35 ± 1.26 32.76 ± 1.22	32.89 ± 1.34 32.89 ± 1.34 32.97 ± 1.46	0.20 ± 1.05 0.41 ± 1.24 0.27 ± 1.22	− 0.14 ± 0.93 − 0.47 ± 1.21 0.21 ± 1.05^#^
Chest	Showering Short bathing Long bathing	33.87 ± 0.76 33.32 ± 1.19* 33.17 ± 1.33*	33.43 ± 1.42 33.41 ± 0.95 32.91 ± 1.42	33.70 ± 1.32 33.90 ± 1.21 33.93 ± 1.03	− 0.44 ± 1.44 0.09 ± 1.21 − 0.26 ± 1.34	0.27 ± 1.08 0.49 ± 1.24 1.03 ± 1.24
Back of the hand	Showering Short bathing Long bathing	30.07 ± 2.59 30.07 ± 2.12 29.09 ± 2.80	32.10 ± 1.30 32.37 ± 1.34 32.31 ± 1.39	29.99 ± 3.49 31.37 ± 2.38 31.65 ± 2.11	2.03 ± 2.61 2.30 ± 2.27 3.23 ± 3.22	− 2.10 ± 3.36 − 1.00 ± 2.32 − 0.67 ± 2.19
Instep	Showering Short bathing Long bathing	28.82 ± 3.76 28.36 ± 3.54 27.90 ± 3.73	30.17 ± 1.88 31.94 ± 1.29** 32.38 ± 1.33**	30.43 ± 3.44 31.86 ± 2.49 32.68 ± 2.35*	1.36 ± 2.70 3.58 ± 3.07** 4.48 ± 3.44**	0.26 ± 2.96 − 0.08 ± 3.03 − 0.30 ± 2.17
DPG	Showering Short bathing Long bathing	− 5.06 ± 4.00 − 4.96 ± 3.93 − 5.27 ± 3.83	− 3.26 ± 1.62 − 1.47 ± 1.73** − 0.53 ± 1.62**^,#^	− 3.27 ± 3.20 − 2.04 ± 2.13 − 1.26 ± 2.35*	1.80 ± 3.30 3.49 ± 3.49 4.74 ± 3.26*	0.01 ± 3.06 − 0.57 ± 3.16 − 0.73 ± 2.46

DPG: distal (instep) to proximal (chest) skin temperature gradient. *A* before bathing, *B* after bathing, *C* before sleep

B-A means B minus A, representing the change from before bathing to after bathing

C-B means C minus B, representing the change from after bathing to before sleep

^*^Significant difference from showering condition, *p* < 0.05

^**^Significant difference from showering condition, *p* < 0.01

^#^Significant difference from short bathing condition, *p* < 0.05

^##^ Significant difference from short bathing condition, *p* < 0.01

Regarding sublingual temperature, significant main effects of bathing condition (*p* = 0.037) and time (*p* < 0.001) and a significant interaction (*p* < 0.001) were seen. In all conditions, sublingual temperature increased significantly from pre-bathing to post-bathing (*p* < 0.001), then decreased significantly until before sleep (*p* < 0.001). Sublingual temperature at pre-bathing was significantly lower with long bathing than with short bathing (*p* = 0.027). After bathing, sublingual temperature was significantly higher with long bathing than with showering or short bathing (*p* < 0.001 each). Before sleep, no significant difference was observed between bathing conditions.

The rise in sublingual temperature associated with bathing (“B-A” in Table 1) differed significantly between conditions (*F* = 40.286, *p* < 0.001), with long bathing showing a significantly greater rise than the other two conditions (*p* < 0.001 each). The fall in sublingual temperature from bathing to sleep (“C-B” in Table 1) differed significantly between conditions (*F* = 8.130, *p* = 0.002), showing a significantly greater fall with long bathing than with short bathing (*p* = 0.005) and a tendency to decrease with long bathing compared with showering (*p* = 0.066).

Regarding skin temperatures, an interaction between bathing condition and time was evident for skin temperatures at the chest (*F* = 3.432, *p* = 0.023), back of the hand (*F* = 3.164, *p* = 0.046), and instep (*F* = 5.510, *p* = 0.003). As a result of post-hoc testing, compared with showering, instep skin temperatures after bathing were significantly higher with both short and long bathing (*p* < 0.001 each), and significantly higher before sleep with long bathing (*p* = 0.010). The increase in skin temperature associated with bathing differed significantly between bathing conditions only at the instep, and the rise in skin temperature at the instep due to bathing (B-A) was significantly higher with both short and long bathing than with showering (short bathing, *p* = 0.007; long bathing, *p* < 0.001). The fall in forehead skin temperature from after bathing to before sleeping (C-B) was significantly higher with long bathing than with short bathing (*p* = 0.041), but no differences in other skin temperatures were noted between conditions.

Regarding DPG, representing the difference in skin temperatures between the instep and chest, main effects of bathing condition (*F* = 7.125, *p* = 0.002) and time (*F* = 23.595,* p* < 0.001) and an interaction between bathing condition and time (*F* = 3.380, *p* = 0.013) were seen. Although no significant differences in DPG were seen before bathing, DPG just after bathing was significantly higher with short and long bathing compared to showering (*p* < 0.001 each), and also significantly higher with long bathing than with short bathing (*p* = 0.044). Furthermore, DPG before sleep was significantly higher with long bathing than with showering (*p* = 0.019). The rise in DPG associated with bathing (B-A) differed significantly between conditions (*F* = 7.644, *p* = 0.001), and the rise was significantly higher with long bathing than with showering (*p* = 0.001). The fall in DPG from bathing to sleep (C-B) did not differ significantly between conditions (*F* = 0.603, *p* = 0.552).

### Thermal sensation before sleep

**Fig. 4:**
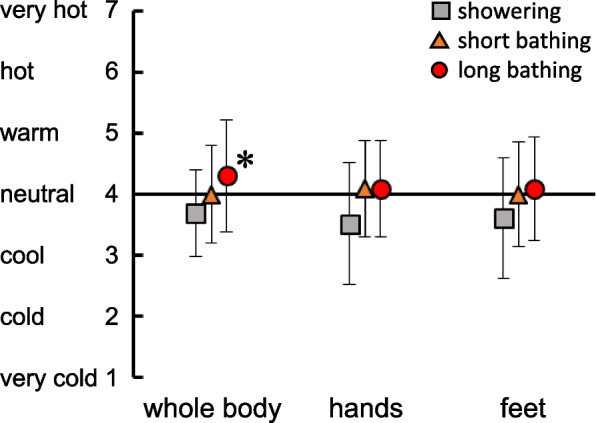
Thermal sensations for the whole body, back of the hand, and feet before going to sleep. Squares, triangles, and circles indicate showering and short and long bathing conditions, respectively. *Significant difference from showering condition, *p* < 0.05

Figure 4 indicates the results from thermal sensations for the whole body, hands, and feet before going to sleep. No differences in thermal sensation at the hands and feet were seen among conditions. However, thermal sensation for the whole body was perceived as higher with long bathing than with showering (*p* = 0.045).

## Discussion

From the results for SL as a sleep index, long bathing showed a significantly shorter time required to fall asleep compared to showering (Fig. 1). In addition, long bathing was significantly superior to showering and short bathing in terms of sleep onset items in Factor II (initiation and maintenance of sleep) of the OSA-MA (Fig. 3), and subjective evaluation (Fig. 2). Previous studies [13, 33] have shown that lowering core temperature before sleep leads to shortening the time required to fall asleep. In fact, core temperature was decreased with long bathing compared to other conditions from after bathing to before sleep (Table 1). Bathing before going to bed is thought to raise the core temperature, which increases peripheral blood flow and skin temperature at the hands and feet, thus promoting heat dissipation and finally a more rapid decrease in core temperature. The increase in core temperature produced by bathing is thought to be effective in promoting a greater decrease in core temperature, subsequently shortening SL.

On the other hand, the decrease in core temperature is the result of heat loss and is not the main cause of induced drowsiness, but rather a higher DPG promotes sleep onset [32, 34]. Tai et al. [11] showed similar results in a longitudinal study of 1094 elderly individuals (mean age, 72.0 years). They found that bathing before bed was significantly associated with shorter actigraphic/self-reported SL and higher DPG. In the present study, DPG before sleep was significantly higher with long bathing compared to showering because peripheral skin temperature (at the instep) was increased relative to trunk skin temperature (chest) (Table 1). To increase DPG, not only a temporary rise in skin temperature after bathing, but also a moderate rise in core temperature as with long bathing causes vasodilation as a thermoregulatory reaction, releasing heat from the periphery. To promote an increase in DPG and thus shorten SL, not only warming the periphery but also raising core temperature to the level observed with long bathing in this study appears effective.

In addition, cold hands and feet before sleep induce to prolong SL [2]. SL is reportedly increased for vasospastic individuals with low skin temperature in the extremities and a tendency to feel cold compared to healthy controls. This is attributed to inefficient heat dissipation from the periphery, preventing an adequate drop in core temperature and a smooth transition to sleep.

These findings suggest that the magnitude of the increase in deep body temperature associated with bathing, such as with long bathing, is closely associated with the ease of falling asleep.

For sleep maintenance items in Factor II of the OSA-MA, long bathing showed significantly higher value than showering (Fig. 3b). Previous studies have reported that passive body heating including bathing shortened WASO [6, 10] and increased slow-wave sleep, which is related to sleep depth [6]. In the OSA-MA, detailed question items related to SM include depth of sleep and waking in the middle of the night. The results of such studies support our results for SM with long bathing. This suggested that greater increases in core body temperature induced improvements in sleep maintenance.

In subjective evaluations, sleep quality was significantly better with long bathing than with showering or short bathing (Fig. 2). Yasuda et al. [19] reported that changing bathing habits from showering to bathing in a bathtub (at 41 °C for 10 min) improved sleep quality, particularly in terms of the feelings of deep sleep and recovery from fatigue. Dorsey et al. [5, 6] also reported that bathing at 40–40.5 °C for 30 min improved subjective sleep quality in women with insomnia, specifically increasing “depth of sleep” and decreasing “restlessness”. Our finding for OSA-MA Factor II in the present study supported those previous studies. Subjective sleep quality, which is evaluated the next morning, would be influenced by not only depth of sleep, but also the above-mentioned improvements in SM and falling asleep. Under the conditions of this study, the increase in core temperature with long bathing was suggested to offer the best improvements in quality of sleep among the conditions tested.

From comparing short and long bathing, differences in the rise in core temperature due to differences in bathing time led to differences in subjective evaluations of sleep. Under the condition of long bathing with a large increase in core temperature, vasodilation was induced, DPG increased, and heat dissipation was promoted, resulting in a large decrease in core temperature. In fact, with long bathing, core temperature was higher after bathing, and decreased significantly from after bathing to before sleep (Table 1).

Matsumoto et al. [35] showed that increase in sublingual temperature with bathing was twice the increase in rectal temperature. Therefore, considering the increase in sublingual temperature with long bathing in this study (0.91 °C), the increase in rectal temperature was speculated to be close to the target increase of 0.5 °C. Previous studies have reported that bathing to the mid-thorax level in 41 °C water for 1.5 h increased rectal temperature by 1.8 ºC [3], and induced delayed sleep onset, disturbed sleep, and frequent awakenings [7]. Although a rise in rectal temperature associated with bathing is necessary to have a positive effect on sleep, an optimal range seems to exist, because excessive rises in rectal temperature adversely affects sleep.

Since this study was conducted in the homes of participants, some limitations must be considered.

First, rectal temperature was not able to be measured as the core temperature, although the bathing time was set based on a predicted rectal temperature change [23]. Since sublingual and rectal temperature responses to bathing differ [35], measurement of rectal temperature may be necessary. Second, we could not continuously measure body temperatures or autonomic nervous indices. Clarification of changes in these indices would help us understand the findings of this study. Evaluation of sleep stages by polysomnography would similarly deepen the understanding of the effects on sleep of increasing body temperature by bathing. Finally, the ages of participants were broad and included participants of both sexes and in each menstrual stage, which may mask differences in the effects between subgroups. Furthermore, it is necessary to evaluate the effect of lifestyle and preference for bathing duration on sleep and its individual variations.

## Conclusions

In this study, field experiments on bathing and sleeping were conducted with participants in a wide range of age groups from young to middle-aged (20 s to 50 s) in actual spaces. Our results showed that bathing 1.5–2 h before bedtime with a relatively large rise in core temperature was more beneficial for falling asleep and sleep quality than bathing with a small rise in core temperature. This study is the first to verify the relationship between the degrees of increase in core temperature associated with bathing and sleep in actual living conditions.

## Data Availability

All relevant data are resented within the paper.

## Abbreviations

ANOVA: Analysis of variance
DPG: Distal-to-Proximal skin temperature Gradient
OSA-MA: Oguri-Shirakawa-Azumi Sleep Inventory, Middle-aged and Aged version
SE: Sleep efficiency
SL: Sleep onset latency
SM: Sleep maintenance
TIB: Time in bed
TST: Total sleep time
WASO: Wake after sleep onset
